# Functional dissection of human targets for KSHV-encoded miRNAs using network analysis

**DOI:** 10.1038/s41598-017-03462-w

**Published:** 2017-06-09

**Authors:** Yu Wang, Yun Lin, Yanzhi Guo, Xuemei Pu, Menglong Li

**Affiliations:** 1 College of Chemistry, Sichuan University, Chengdu, 610064 P.R. China; 2 Department of Editor, Sichuan Academy of Medical Sciences and Sichuan Provincial People’s Hospital, Chengdu, 610072 P.R. China

## Abstract

Kaposi’s sarcoma-associated herpesvirus (KSHV) is the etiological agent of Kaposi’s sarcoma, primary effusion lymphoma and multicentric Castleman’s disease, etc. In this study, we firstly systematically constructed the KSHV-encoded miRNA-regulated co-expressed protein-protein interaction network (CePPIN), which display the biological knowledge regarding the mechanism of miRNA-regulated KSHV pathogenesis. Then, we investigated the topological parameters for the proteins in CePPIN, especially for those miRNA targets and we found that cellular target genes of KSHV-encoded miRNAs tend to be hubs and bottlenecks in the network. Then the GO and KEGG pathway analysis suggests that miRNA targets are involved in various cellular processes mostly related to immune regulate and cell cycle. Enrichment analysis was also performed to identify the six important functional modules which are proven to be highly related to KSHV pathogenesis. Finally, difference analysis of common targets and specific targets shows that two kinds of targets are different in terms of both topological properties and enriched functions, thus we can extrapolate that the functions of KSHV-encoded miRNAs can be also classified into two generic groups, one can act as functional mimics of some oncogenic human miRNAs which contribute to tumorigenesis and the other can contribute to maintaining viral survival.

## Introduction

microRNAs (miRNAs) are a set of small non-coding RNA molecules with a length of 21~24 nucleotides and function as major gene expression regulators at the posttranscriptional level in eukaryotic cells^1^. However, some viruses also express miRNAs in host cells and 5 virally-encoded miRNAs were firstly discovered by Pfeffer *et al*. in human B cells after infection with the herpesvirus Epstein–Barr virus (EBV)^2^. Since then, more than 200 mature miRNAs have been identified in all herpesviruses, except for Varicella Zoster virus^3, 4^. miRNAs are complementary to specific sequence motifs in the 3′ untranslated regions (UTRs) of their target mRNAs and negatively regulate gene expression at the post-transcriptional level by inhibiting translation or promoting mRNA degradation, based on the degree of complementary base pairing between the miRNA and mRNA. More and more evidence have revealed that miRNAs are involved in diverse physiological processes, such as development^5^, cell proliferation and differentiation^6, 7^, apoptosis^8^ and a variety of pathological conditions^9, 10^.

The human tumor virus Kaposi’s sarcoma-associated herpesvirus (KSHV), also called human herpesvirus 8 (HHV8), is the etiological agent of Kaposi’s sarcoma (KS), the most common malignance in AIDS patients, primary effusion lymphoma (PEL) and some types of multicentric Castleman’s disease (MCD)^11–13^. KSHV has been confirmed to encode at least 25 mature miRNAs^14–17^. Currently 16 experimentally confirmed KSHV miRNAs and their targets are available, but knowledge about their functions remains scarce since only a small number of mRNA targets have been experimentally validated. Study on the function of viral miRNA targets would contribute to further dissecting the role of viral miRNAs in viral-host interactions, and then might contribute to an elucidation of virus infection mechanism and potential identification of antiviral targets. Characterizing the direct targets of miRNAs has been widely employed to map miRNAs to biological pathways and diseases^18–20^. However, the regulatory effects of a miRNA are not only due to the direct RNA-induced silencing complex (RISC)-dependent targeting. Both direct and indirect targets are integral components of gene regulatory networks and should be together considered in functional analysis. In addition, proteins that physically associate with direct targets to function together in a complex may also be affected. Hsu *et al*.^21^ named the direct targets of miRNAs “L0 proteins” and the interacting partners of these targets “L1 proteins” in their protein-protein interaction network (PPIN). By comparing four topological parameters, namely degree, betweenness centrality, clustering coefficient and closeness centrality of L0 proteins and L1 proteins jointly with randomly selected proteins, it reveals that miRNA-regulated targets and their interacting partners jointly show significantly higher connectivity and modularity than randomly selected proteins, even than direct targets alone. In their following work^22^, they demonstrate that L0 proteins and L1 proteins together also show a stronger functional relationship than L0 proteins alone. They further named the network formed by L0 proteins and L1 proteins the co-expressed protein-protein interaction network (CePPIN). Recent studies have shown that integrating miRNA targets and PPIN makes it more efficient to dissect the regulation rules of miRNAs^23^.

In this study, to further understand the regulation rules of KSHV-encoded miRNAs, we constructed the miRNA-regulated CePPIN for 16 KSHV miRNAs and 109 experimentally validated human target genes. We firstly investigated the topological properties of the proteins in CePPIN, especially for those miRNA targets. Subsequently we analyzed the function of the proteins in CePPIN from multi-perspectives including the GO and KEGG pathway analysis and functional module extraction. Finally, to further explore difference between common targets and specific targets, we investigated the topological properties and functional annotation for genes targeted by both KSHV-encoded miRNAs and human miRNAs and genes only targeted by KSHV-encoded miRNAs, respectively. Consequently, these results provide new clues for elucidating the regulation rules of KSHV-encoded miRNAs in viral-host interactions.

## Result and Discussion

### Statistical analysis between KSHV-encoded miRNAs and their human targets

**Figure 1:**
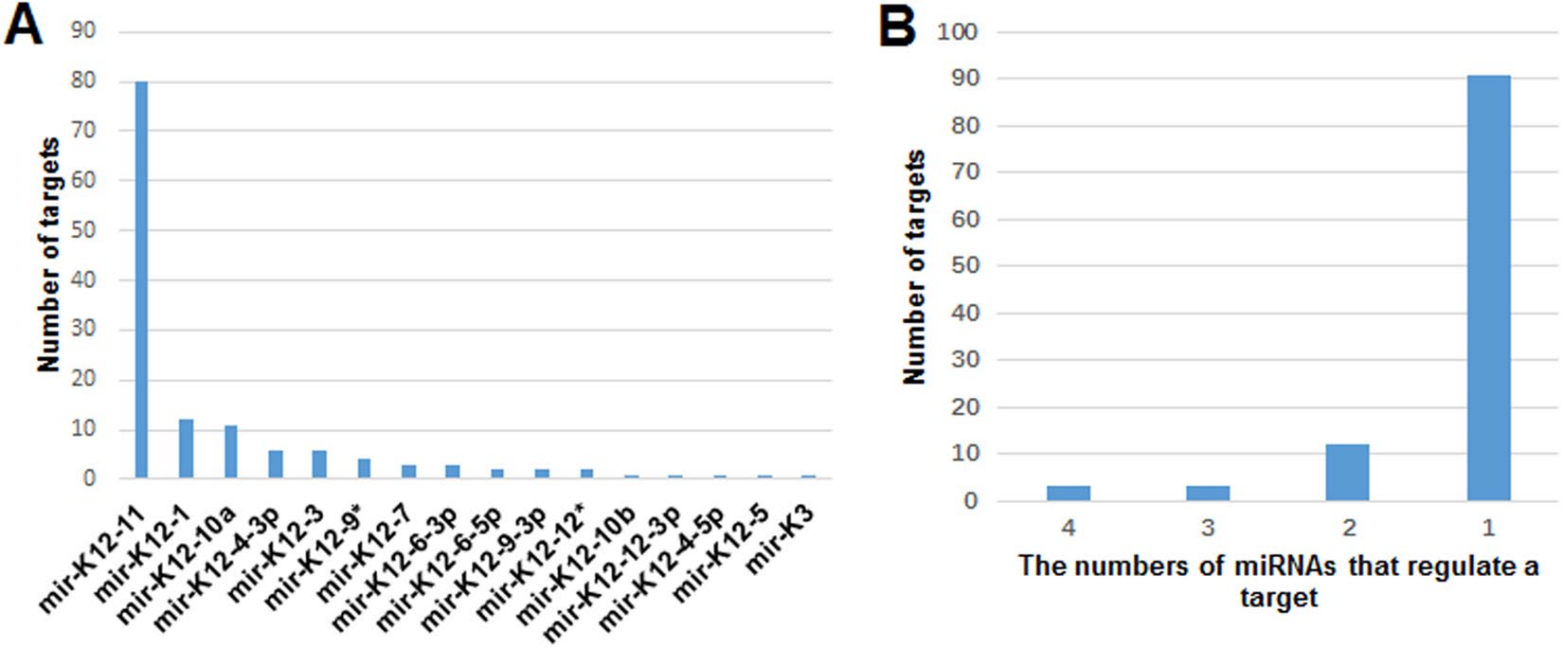
Statistical analysis of KSHV miRNAs and their human targets. (**A**) Number of human targets that each KSHV miRNA regulates. (**B**) Number of targets regulated by different numbers of KSHV miRNAs.

For 16 KSHV miRNAs and 109 human targets, we counted the number of human targets that each KSHV miRNA regulates. As shown in Fig. 1A, almost three quarters (80) of the 109 targets were regulated by miR-K12-11. miR-K12-11 is the ortholog of host hsa-miR-155 and these miRNAs have identical seed sequence. Has-miR-155 is a multifunctional miRNA that is important in immunity, hematopoiesis, inflammation, and oncogenesis. *In vitro* studies have shown that both miRNAs can regulate a common set of cellular target genes, suggesting that KSHV miR-K12-11 may mimic hsa-miR-155 function^36, 37^. Moreover, the KSHV-encoded miRNAs that regulate relatively more human targets, such as miR-K12-10a and miR-K12-3, are also the ortholog of host hsa-miR-142-3p and hsa-miR-23^38^, respectively. The results of sequence alignment between KSHV miRNAs and human miRNA^36–38^ also suggests that some KSHV miRNAs might act as functional mimics of some oncogenic human miRNAs which contribute to tumorigenesis.

On the other hand, we counted the number of targets regulated by different numbers of KSHV miRNAs. As can be seen from Fig. 1B, only 6 human targets are regulated by 3 or 4 kinds of KSHV miRNAs. They are BCLAF1 (4 miRNAs), MAF (4 miRNAs), THBS1 (4 miRNAs), WEE1 (3 miRNAs), BACH1 (3 miRNAs) and CDKN1A (3 miRNAs) respectively and they are all protein coding genes. BCLAF1 (Bcl-2-associated factor), a pro-apoptotic protein, was identified as a target of miR-K12-5, -9, -3 and -10b. The repression of BCLAF1 by KSHV miRNAs enabled the cells to overcome etoposide-induced caspase activation^39^. THBS1 (thrombospondin 1) was identified as a target of miR-K12-1, -3, -6-3p and -11 and it is a protein of the TGF-β pathway. The TGF-β pathway confers a strong anti-proliferative phenotype to many epithelial and endothelial cells and hence, it is presumable that KSHV has developed mechanisms to escape from this inhibition^40^. BACH1 (BTB and CNC homology 1), a transcription repressor involved in the regulation of genes regulating cell cycle and oxidative stress^41^. Down-regulation of the LEC-specific transcription repressor, MAF, by KSHV miRNAsmiR-K12-6 and miR-K12-11, was shown to induce the expression of several BEC-specific markers like CXCR4, thereby contributing to transcriptional reprogramming^42^. KSHV miR-K12-1 down-regulated the levels of the cyclin-dependent kinase inhibitor, CDKN1A (p21), thereby allowing the infected cells to overcome p21-mediated cell cycle arrest^43^. WEE1 is a key regulator of cell cycle progression. Down-regulated of WEE1 might decrease the efficiency of DNA safeguard mechanisms thus enhance the rate of spontaneous mutations^44^. These six targets are all important to KSHV infection. It is possible that only a relatively small subset of KSHV-miRNAs targets provides a strong selective advantage to KSHV, while many other miRNAs-targets interactions could be largely inconsequential to KSHV biology. Such particularly important targets should include those with multiple KSHV miRNA binding sites, for example CDKN1A (p21) or Wee1.

### Construction of CePPIN for human targets of KSHV miRNAs

**Figure 2:**
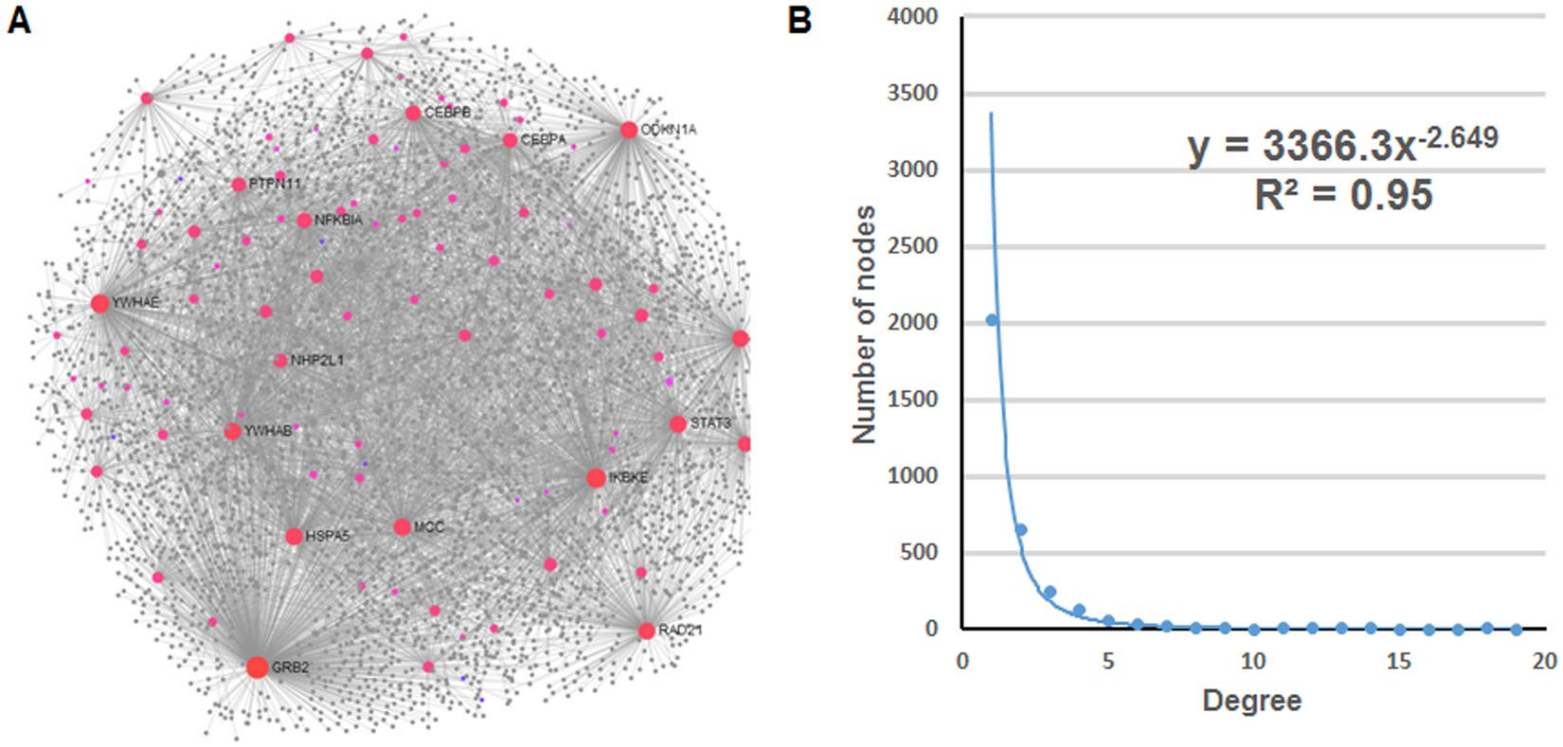
The visualization of KSHV miRNA-regulated CePPIN. (**A**) MiRNA-regulated CePPIN generated by Network Analyst. Red nodes are miRNA targets and grey nodes are non-targets. The 16 nodes in bigger size and with labels have the highest degrees in CePPIN. (**B**) Degree distribution of proteins in CePPIN.

We used the 109 human targets as source to compile the human protein-protein interaction (PPI) information from InnateDB^26^. Overall, we obtained 3281 proteins (100 targets and 3181 co-expressed proteins) and 5730 PPIs as the original dataset of CePPIN. 9 targets were discarded for lacking the interactome information. The visualization of CePPIN is shown in Fig. 2A. A scale-free network is a network whose degree distribution follows a power law. As we can see from Fig. 2B, the degree distribution of CePPIN follows a power law. Small world networks are those that have a relative small mean path length but high clustering coefficient. Since the average shortest path length of all the nodes in CePPIN is about 3.5124 and the average clustering coefficient is 0.0823, so we can see that CePPIN presents the properties of small-world and scale-free network, which indicates that a small number of proteins called hubs directly regulate most of other members in the network. This network is more robust against random variation but more fragile against some aberration of the hubs compared with random network. In Fig. 2A, the bigger red nodes with labels are the top sixteen-ranked proteins by their degrees in CePPIN. Their degrees are all higher than 100 and they are all miRNA targets. For example, NFKB Inhibitor Alpha (NFKBIA) is down-regulated by kshv-mir-K12-1, leading to NFKB activation and maintenance of latency. The cyclin-dependent kinase inhibitor p21(CDKN1A) down-regulated by KSHV miR-K12-1, allowing the infected cells to overcome p21-mediated cell cycle arrest^43^. More information of the sixteen proteins is also shown in Table 1. It can also be seen explicitly that the overwhelming majority of miRNA targets have more interactions than other nodes and may play an important role in CePPIN.

**Table 1:** Detailed information of the top 16 proteins by degree in CePPIN.

Symbol	Name	Degree	Betweenness centrality
GRB2	Growth Factor Receptor Bound Protein 2	640	0.2997
IKBKE	Inhibitor of Kappa Light Polypeptide Gene Enhancer In B-Cells, Kinase Epsilon	366	0.1383
YWHAE	Tyrosine 3-Monooxygenase/Tryptophan 5-Monooxygenase Activation Protein Epsilon	310	0.1086
CDKN1A	Cyclin Dependent Kinase Inhibitor 1A	249	0.0901
HSPA5	Heat Shock Protein Family A (Hsp70) Member 5	236	0.0789
MCC	Mutated in Colorectal Cancers	231	0.0654
YWHAB	Tyrosine 3-Monooxygenase/Tryptophan 5-Monooxygenase Activation Protein Beta	227	0.0778
STAT3	Signal Transducer And Activator Of Transcription 3	222	0.0751
RAD21	RAD21 Cohesin Complex Component	217	0.0734
FOS	Fos Proto-Oncogene, AP-1 Transcription Factor Subunit	214	0.0813
CEBPB	CCAAT/Enhancer Binding Protein Beta	155	0.0452
RXRA	Retinoid X Receptor Alpha	151	0.0532
NFKBIA	NFKB Inhibitor Alpha	142	0.0380
CEBPA	CCAAT/Enhancer Binding Protein Alpha	133	0.0365
PTPN11	Protein Tyrosine Phosphatase, Non-Receptor Type 11	122	0.0344
NHP2L1	NHP2-like protein 1	107	0.0322

### Topological analysis for KSHV miRNA-regulated CePPIN

**Figure 3:**
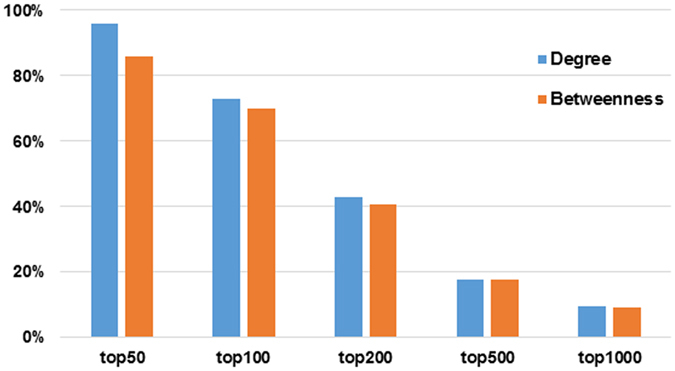
Topological properties of miRNA-regulated CePPIN. The ratios of miRNA targets in top 50, 100, 200, 500, 1000 nodes sorted by degree and betweenness centrality respectively.

Four important topological parameters were computed for all nodes in CePPIN, namely degree, betweenness centrality, clustering coefficient and closeness centrality, respectively. Table 2 shows the topological parameters of all nodes and miRNA targets respectively in CePPIN. Since the range of the four topological parameters are very wide, the deviation values are very high. For example, among all proteins, the minimum degree is 1 and the maximum is 644, so the deviation is as high as 19.0992. In fact, the four topological parameters are all nonnegative number. We can see that the average degree and betweenness of miRNA targets are much higher than those of all nodes and the p-values are much lower than 0.05 by t-test. Degree and betweenness centrality are considered to be the best predictor of the essentiality of a node for network robustness, cooperation and communication and closeness centrality is a measure of centrality in a network. Thus, we can conclude that miRNA targets are essential in the CePPIN. At the same time, clustering coefficient of miRNA targets which quantifies the cohesiveness of the neighborhood of a node are much lower, indicating that miRNA targets are dispersed in the network. We further counted the percentage of targets in top 50, 100, 200, 500 and 1000-ranked nodes by their degrees and betweenness centralities respectively in the whole CePPIN. As we can see from Fig. 3, most of the targets have the highest degree and betweenness centrality in CePPIN, indicating these targets tend to be hub or bottleneck proteins in the network. Among the 100 miRNA targets, there are 88 ones with degrees higher than the average value of all proteins and 81 with betweenness centrality higher than the average value of all proteins. Owing to the existence of these miRNA targets, the CePPIN remains robust against random attack, but it is more sensitive to even slight changes of miRNA expression and more efficient to transfer the changes to other proteins, which can make a global influence on the whole PPIN.

**Table 2:** Topological parameters of all nodes and miRNA targets in CePPIN.

	Degree	Betweenness centrality	Clustering coefficient	Closeness centrality
All proteins	3.4928 ± 19.0992	7.75 × 10^−4^ ± 8.02 × 10^−3^	0.0823 ± 0.2410	0.2847 ± 0.0266
miRNA targets	57.5050 ± 93.6908	0.0186 ± 0.0382	0.0156 ± 0.0501	0.3373 ± 0.0349

### Functional analysis of human targets of KSHV-miRNAs

**Figure 4:**
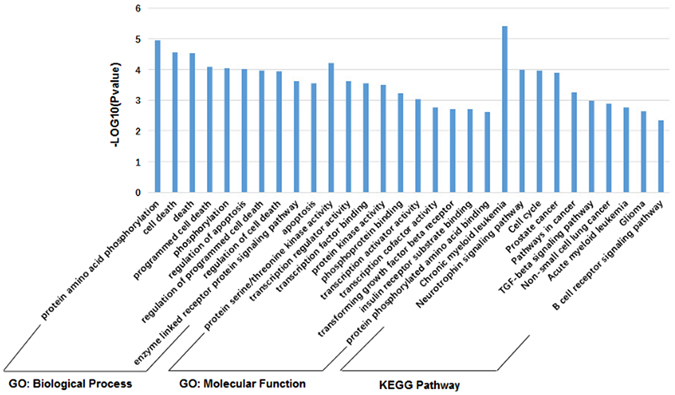
Functional analysis of miRNA targets in CePPIN. The selected most enriched GO: biological process, GO: molecular function and KEGG pathway terms (p-value < 0.05) for all miRNA targets.

We performed gene ontology (GO) and KEGG pathway analysis on all 109 miRNA targets. Totally, 83 GO: biological process (GO: bp), 27 GO: molecular function (GO: mf) and 19 KEGG pathway terms (p-value < 0.05) were found for these targets. Figure 4 shows the selected top 10 enriched GO terms and KEGG pathway terms for these targets. We can see that most of these enriched biological process terms are associated with oncogenesis, including cell death, apoptosis, transcriptional regulation and so on. For example, apoptosis is significantly enriched by targets of KSHV miRNAs, and inhibiting apoptosis contributes to maintaining KSHV survival. Several KSHV miRNAs can inhibit apoptosis such as miR-K5, -K9, and -K10a/b by targeting Bcl-2 associated factor (BCLAF1), a pro-apoptotic protein. On the other hand, all the molecular function terms are about the kinase activity and transcription factor binding, which are all molecular mechanisms related to cell cycle. KSHV genomes replicate once per cell cycle in latent cells and depends fully on cellular replication machinery for replication and genome maintenance, so cell cycle control regulated by KSHV miRNAs is essential for KSHV genomes replication. As for KEGG pathway, most of the enriched pathways are cell cycle and some cancer-related pathways such as pathways in cancer, TGF-β and so on. There are also some immune-related pathways such as B cell receptor signaling pathway included. These enriched GO terms and KEGG pathways are consistence with the conclusion that KSHV-encoded microRNAs tend to target immune modulatory genes and cell cycle control genes^45^.

**Figure 5:**
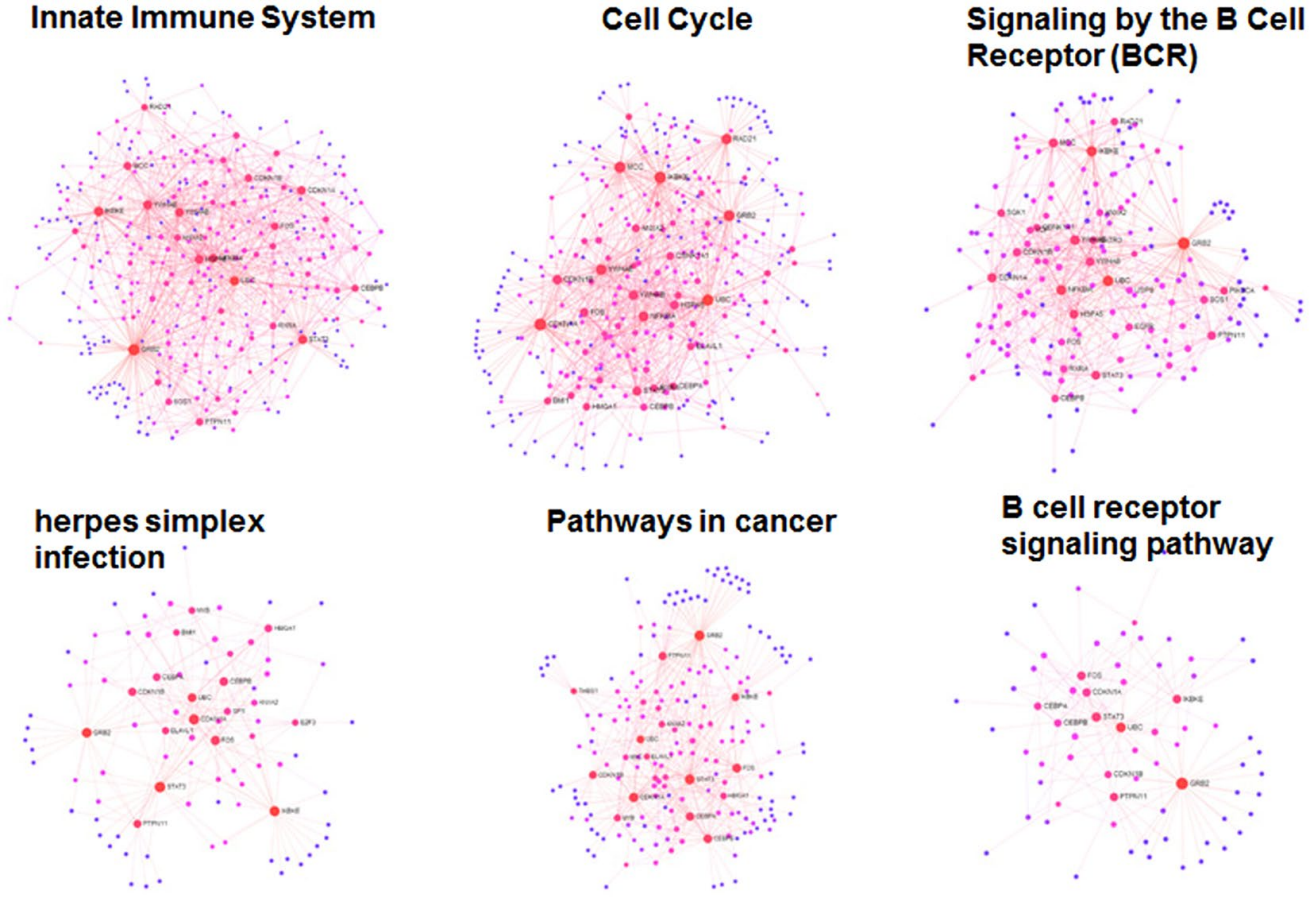
Six extracted functional modules from CePPIN by using enrichment analysis.

Furthermore, we identified and extracted functional modules from the KSHV miRNA-regulated CePPIN by using enrichment analysis in Reactome^46^ database with Network Analyst. As a result, six network modules of highly-interacting groups of genes were identified and extracted, namely innate immune system, cell cycle, signaling by the B Cell Receptor (BCR), herpes simplex infection, pathways in cancer and B cell receptor signaling pathway, respectively. The sub-networks of these six extracted functional modules were shown in Fig. 5. All these six important functional modules are proven to be highly related to KSHV pathogenesis. For example, I-kappa-B kinase epsilon (IKKɛ, gene symbol IKBKE) is a target of KSHV-miR-K12-11 in functional module innate immune system. KSHV-miR-K12-11 is critical for the modulation of Type I interferon (IFN) signaling, the principal response mediating antiviral innate immunity and acts through targeting IKKɛ. Importantly, expression of miR-K12-11 attenuated IFN signaling by decreasing IKKɛ-mediated IRF3/IRF7 phosphorylation and further inhibiting the activation IKKɛ-dependent IFN stimulating genes (ISGs), allowing miR-K12-11 suppression of antiviral immunity^47^. Therefore, it can be asserted that miR-K12-11 can contribute to maintenance of KSHV latency by targeting IKKɛ.

### Comparative analysis on common targets and specific targets

**Figure 6:**
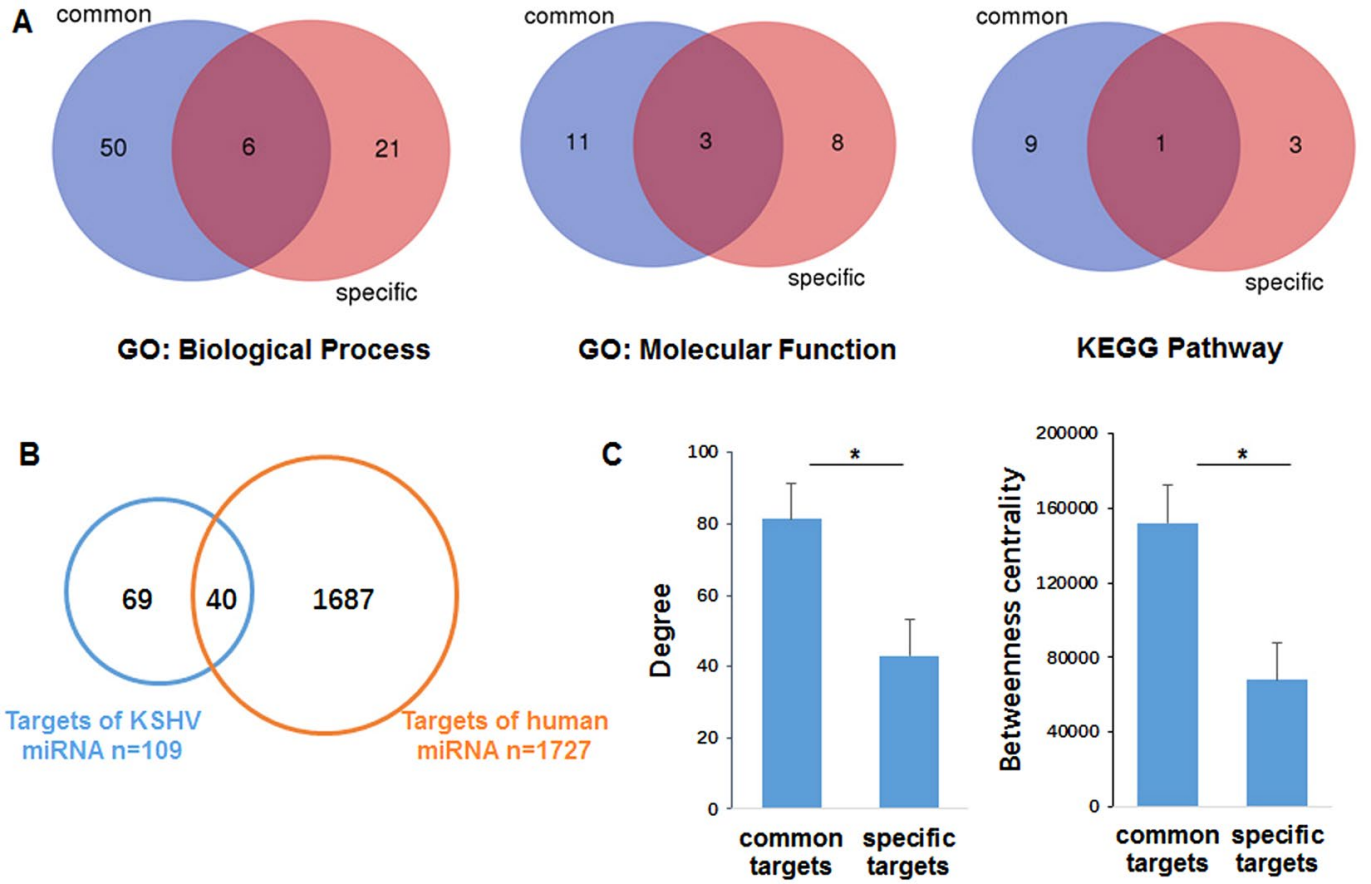
Difference analysis of common targets and specific targets. (**A**) The number and overlaps of GO: biological process, GO: molecular function and KEGG pathway terms (p-value < 0.05) for common targets and specific targets. (**B**) Overlaps of targets between KSHV miRNAs and human miRNAs. Blue represents KSHV miRNAs and orange represents human miRNAs. (**C**) Difference analysis of common targets and specific targets in terms of degree and betweenness centrality (t-test, *p < 0.05).

**Figure 7:**
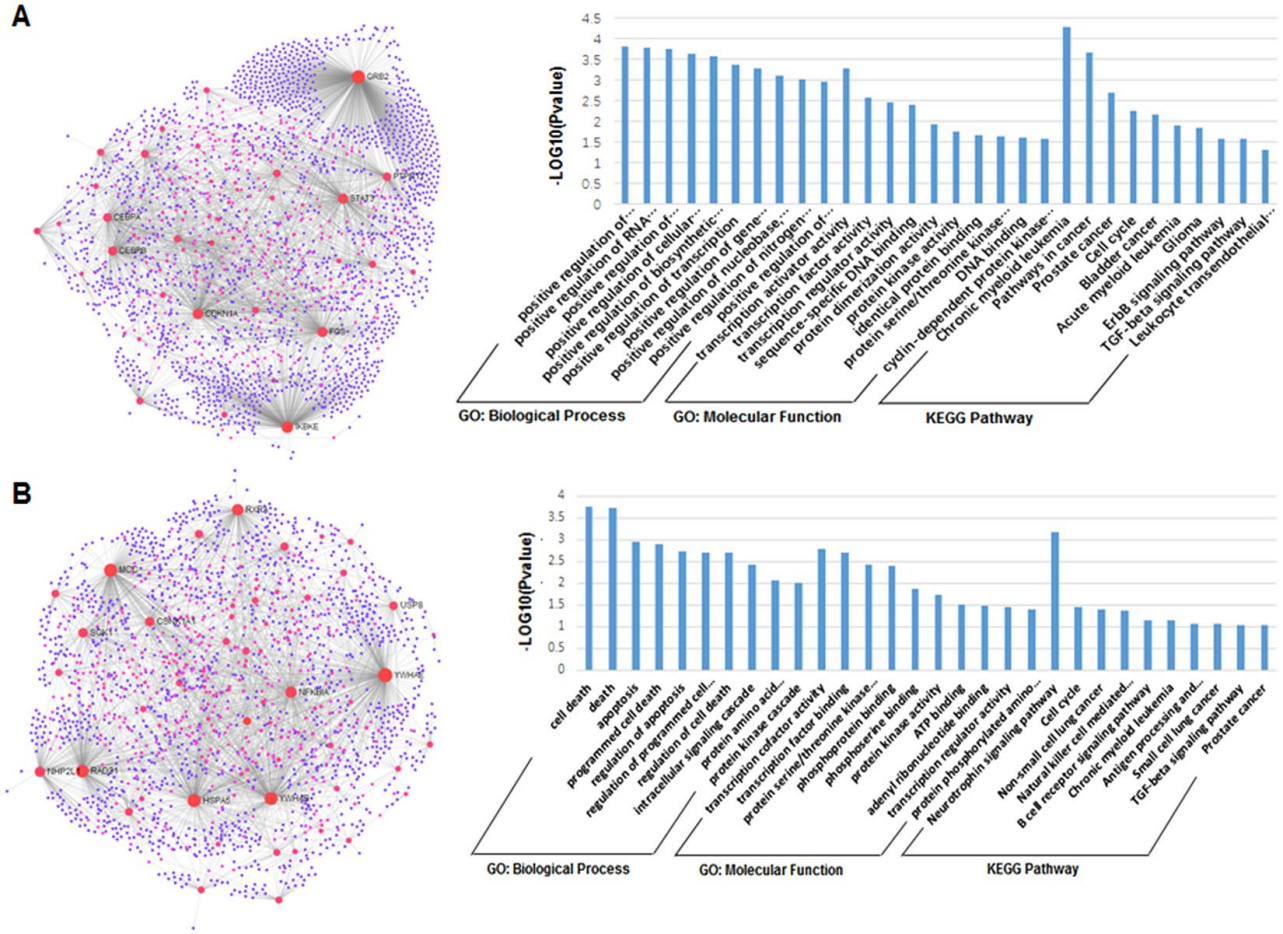
The visualization and functional analysis of KSHV miRNA-regulated CePPIN on common targets and specific targets. (**A**) CePPIN and functional analysis on common targets. (**B**) CePPIN and functional analysis on specific targets.

As shown in Fig. 6B, through mapping the 109 human targets of KSHV-encoded miRNAs to 1727 targets of human miRNAs, we found an overlap of 40 genes, which indicating that a common set of targets are co-regulated by both some KSHV miRNAs and human miRNAs. We called the genes targeted by both KSHV miRNAs and human miRNAs as the common targets and the genes only specifically targeted by KSHV as the specific targets. To further investigate the difference between these two kinds of targets, we constructed miRNA-regulated CePPIN for the common targets (Fig. 7A) and specific targets (Fig. 7B), respectively. After that, we also performed gene ontology (GO) and KEGG pathway analysis on these two kinds of targets respectively. Figure 6A displays the number of enriched GO terms and KEGG pathway terms for common targets and specific targets and the number of overlap terms between them. It demonstrates that few common enriched GO terms and KEGG pathways can be found between the common targets and specific ones. In addition, we also performed gene ontology (GO) and KEGG pathway analysis on the remaining human targets and the KSHV targets. Compared to 113 GO: biological process, 32 GO: molecular function and 60 KEGG pathway terms of the remaining 1687 targets, only a minor portion of them are overlapped with those of the 109 KSHV targets, including 17 GO: biological process, 10 GO: molecular function and 14 terms KEGG terms (p-value < 0.05). It also shows that a very few common enriched GO terms and KEGG pathways can be found between the remaining human targets and the KSHV targets.

Moreover, we computed degree and betweenness centrality for common targets and specific targets in the whole miRNA-regulated CePPIN. Figure 6C shows the degree and betweenness centrality of common targets and specific targets. We can see that common targets and specific targets have significant difference in both degree and betweenness centrality by t-test (p-value < 0.05). These findings also suggest that KSHV miRNAs can be also classified into two generic groups, in which one can act as functional mimics of some oncogenic human miRNAs that contribute to tumorigenesis and the other could contribute to its own latent viral replication.

## Conclusion

This work focuses on dissecting the regulation role of KSHV-encoded miRNAs through topological and functional analysis of human targets in miRNA-regulated CePPIN. We found that miRNA targets are essential in CePPIN with high degrees, betweenness centralities, closeness centralities and low clustering coefficients, thus they can make a global influence on the whole PPIN. Moreover, these targets are involved in various cellular processes especially related to immune regulate and cell cycle control, which contributes to KSHV immune escape and maintaining KSHV survival. In addition, the comparisons between the two sub-CePPINs for common targets and specific targets on the topological and function analysis indicate that significant difference are found in terms of both topological properties and enriched functions. So we can speculate that KSHV miRNAs mainly have two roles in viral-host interactions. One role is acting as functional mimics of some oncogenic human miRNAs which contribute to tumorigenesis and the other can facilitate to maintaining KSHV survival. Overall, the functional dissection of human targets for KSHV-encoded miRNAs in our work provides helpful information for further understanding the role of KSHV-encoded miRNAs in viral-host interaction from the perspective of the co-expressed protein-protein interaction network and hopefully may facilitate the researches on the current prevention and treatment of KSHV-induced cancers.

## Material and Methods

### Dataset of miRNAs and targets

The data of KSHV miRNAs and the corresponding human targets were downloaded from VmiReg database^24^. This database collects experimentally validated and predicted target genes of viral miRNAs. Here, we focused on experimentally validated human targets only and totally, we collected 16 KSHV miRNAs and the corresponding 109 human targets. In addition, an experimentally validated dataset of 1727 human targets of human miRNAs was also collected from the work of Shao *et al*.^24^.

### PPIN construction and visualization

In our study, the construction and visualization of PPIN was generated by Network Analyst^25^ which contains the initial human PPI data from InnateDB^26^. In addition, NetworkAnalyst not only uses a comprehensive high-quality protein-protein interaction (PPI) database based on InnateDB but also contains manually curated protein interaction data from published literature as well as experimental data from several PPI databases including IntAct, MINT, DIP, BIND, and BioGRID. We mapped 109 human targets of 16 KSHV miRNAs to the PPI data to find the co-expressed proteins of these targets. 100 human targets and their co-expressed proteins were extracted from the original data, since 9 targets were discarded for lack of interactome information. Then, we used selected PPIs to construct the virally-encoded miRNA-regulated CePPIN for the human targets of the KSHV miRNAs. The topological parameters of CePPIN were computed using the Cytoscape (version 3.2.0)^27^ plugin Network Analyzer.

### Topological parameters of PPIN

Topological analysis of biological networks is a topic of great interest in the field of current bioinformatics and systems biology. It provides quantitative insight into biological systems, especially for PPIN. It is a more effective strategy to identify important molecules, analyze functional modules or pathways and further uncover the mechanism of molecular interaction^28^, even to predict new molecular interaction^29, 30^.

In this study, four important topological parameters were used to describe the characterization of the nodes in CePPIN, including degree, betweenness centrality, clustering coefficient and closeness centrality respectively. Degree denotes the number of edges linked to the specified node in the network. In scale-free network, the nodes with degrees much higher than the average degree of the whole network are usually defined as hubs^31^. Betweenness centrality measures the number of non-redundant shortest paths going through a node. Studies have reported that nodes with high betweenness centralities called bottlenecks might act as an important link between two groups of nodes^32^. In most cases, nodes with high degrees tend to have high betweenness centralities and the two parameters are considered to be the best predictor of the essentiality of a node for network robustness, cooperation and communication. Clustering coefficient is defined as the ratio between the number of edges linking adjacent nodes and the total number of possible edges among them. Closeness centrality can be calculated as the reciprocal of the average shortest path length between a node and any other node in network. Nodes with higher closeness centralities are closer to other nodes in location and can also be more efficient to transmit information.

### Functional analysis

To understand the underlying biological processes and pathways of downstream PPIs, we used the DAVID (The Database for Annotation, Visualization and Integrated Discovery)^33^ online tool (http://david.abcc.ncifcrf.gov/) to conduct PPI enrichment analysis. Investigations were implemented on GO (Gene Ontology)^34^ biological process, GO molecular function and KEGG (Kyoto Encyclopedia of Genes and Genomes)^35^ pathways at the significant level (p-value < 0.05). Furthermore, we also identified and extracted functional modules from the KSHV miRNA-regulated CePPIN by using enrichment analysis with Network Analyst^25^.
